# German emergency department measures in 2018: a status quo based on the Utstein reporting standard

**DOI:** 10.1186/s12873-021-00563-8

**Published:** 2022-01-11

**Authors:** Florian Wallstab, Felix Greiner, Wiebke Schirrmeister, Markus Wehrle, Felix Walcher, Christian Wrede, Kirsten Habbinga, Wilhelm Behringer, Dominik Brammen

**Affiliations:** 1 Department of Trauma Surgery, Otto von Guericke University Magdeburg, Magdeburg, Germany; 2 Department of Anesthesiology and Intensive Therapy, Otto von Guericke University Magdeburg, Magdeburg, Germany; 3 Department of Emergency Medicine, Hospital Berlin-Buch, Berlin, Germany; 4 Department of Interdisciplinary Emergency Medicine, Pius-Hospital, Oldenburg, Germany; 5 Department of Emergency Medicine, Medical University of Vienna, Vienna, Austria

**Keywords:** Emergency medicine, Emergency department structures, Utstein reporting standard, Germany

## Abstract

**Background:**

Compelling data on clinical emergency medicine is required for healthcare system management.

The aim of this survey was to describe the nationwide status quo of emergency care in Germany at the healthcare system level using the Utstein reporting template as the guideline to measure the data collected.

**Methods:**

This cross-sectional survey collected standardized data from German EDs in 2018. All 759 of the EDs listed in a previously collected ED Directory were contacted in November 2019 using the online-survey tool SoSci Survey.

Exclusively descriptive statistical analyses were performed. Absolute as well as relative frequencies, medians, means, ranges, standard deviations (SD) and interquartile ranges (IQR) were reported depending on distribution.

**Main Results:**

A total of 150 questionnaires of contacted EDs were evaluated (response rate: 19.8%). Hospitals had a median of 403 inpatient beds (*n*=147). The EDs recorded a median of 30,000 patient contacts (*n*=136). Eighty-three EDs (55%) had observation units with a median of six beds. The special patient groups were pediatric patients (< 5 years) and older patients (> 75 years) with a median of 1.7% and 25%, respectively. Outpatients accounted for 55%, while 45% were admitted (intensive care unit 5.0%, standard care unit 32.3%, observation unit 6.3%) and 1.2% transferred to another hospital.

**Conclusions:**

The use of the Utstein reporting template enabled the collection of ED descriptive parameters in Germany. The data can provide a baseline for upcoming reforms on German emergency medicine, and for international comparisons on admission rates, initial triage categories, and patient populations.

## Background

Providing care to a wide spectrum of emergency patients is a key task in healthcare systems. In Germany, emergency care is provided by emergency medical services, emergency departments and physicians in private practice. There are no official ED patient count statistics available for Germany. Published data showed increasing patient numbers in EDs, accompanied by ongoing professionalization of emergency medicine [1–5]. But those estimates are based on data on inpatient claims with an administrative emergency definition [6] or outpatient data missing relevant patient groups [7]. There were 1,864 hospitals with 1,065 emergency departments (EDs) in 2018 [8, 9]. However, detailed data regarding the organization and performance of EDs in Germany are scarce [10], partly attributed in part due to the missing specialty for emergency medicine in Germany. Therefore, robust data as a basis for political decision-making are lacking as well. The aim of this survey was to describe a nationwide status quo of care in emergency departments in Germany using the SocSci Survey tool with focus on demographic patient data, ED structure and process indicators by using the Utstein template. This "template for uniform reporting of emergency department measures, consensus according to the Utstein method" was developed by Hruska et al. to enable a comparative description of individual EDs in research publications [11] and was adapted to the German ED and hospital structures.

## Methods

### Study design

A cross-sectional online survey was conducted to collect key data from German EDs for the reference year 2018. After translation into German, the Utstein reporting template was used to develop a questionnaire. This was adapted and consented by clinical and methodological experts.

In November 2019, all 759 ED chairs listed in a previously collected ED directory were invited via email to participate in the survey using the online-tool SoSci Survey (SoSci Survey GmbH, Munich, Germany). Participation in the survey was anonymous and voluntary. The survey ended in January 2020, and lasted for two months, with three reminders being sent out periodically.

The study was approved by the Ethics Committee of the Otto von Guericke University at the Faculty of Medicine, Magdeburg, Germany (identification number 131/19).

### Questionnaire and adaption of the Utstein template

The questionnaire included 19 questions according to ED workflow with minor modifications of the Utstein template to accommodate German conditions. Data were collected on ED structures, processes, and patient characteristics. The measure of acute care beds per 1,000 inhabitants was replaced with an intensive care bed count. The time to first provider was defined as time to first physician because other professions listed in the Utstein template were not common in Germany until 2021. As there was no specialty for emergency medicine established in Germany, the question of coverage by emergency medicine specialists was excluded. Questions about the proportion of disposition of non-hospitalized patients, proportion of patients until age of 18, and the German federal state of hospital location were added. Clinical care hours were collected separately and cumulatively in hours per 100 cases by occupational group.

To allow for the distinction between a lack of data or questions not answered, participants could respond to each question with "unknown".

### Inclusion criteria

Only EDs that visited all pages of the questionnaire were included in the analysis. In addition, information on hospital beds or ED cases had to be entered as a minimum requirement.

### ED directory

There are 1,065 EDs in Germany [8]; however, the official hospital directory [9] with 1,864 hospitals contains neither information about ED existence nor ED contact data.

To compensate for this, a proprietary ED directory was compiled on behalf of the German Interdisciplinary Association of Critical Care and Emergency Medicine (DIVI) and the German Interdisciplinary Society for Emergency and Acute Medicine (DGINA) containing contacts from 759 EDs at survey time. The representativeness of the hospitals was estimated by comparison with official hospital directory data [9] containing 1,864 hospitals.

### Statistics

After data collection was completed, surveys that met the inclusion criteria were analyzed. Descriptive statistical analyses were performed using Excel 2016 (Microsoft Corp., Redmond, USA) and SPSS 26.0 (IBM Corp., New York, USA). Absolute and relative frequencies, medians, means, ranges, standard deviations (SD) and interquartile ranges (IQR) were reported. The analysis of the structural parameters was carried out in subgroups according to official hospital directory bed count categories (≤ 399 beds, 400-799 beds, ≥ 800 beds). In the context of the descriptive characterization of EDs in Germany, no inductive statistical tests were performed. The valid dataset of the various parameters differed due to omission of missing data (i.e., no responses or "unknown" and "no information").

## Results

### Response rate and representativity

After the interview period, 154 questionnaires were completed. Of these, 150 were evaluated, as four questionnaires did not meet the inclusion criteria. In relation to contacted EDs, the response rate of this study was 19.8%, representing 14.1% of the 1,065 hospitals with EDs [8] in Germany. The median number of hospital beds in this study was higher than the German average [9] of all 1.864 hospitals including non-ED hospitals. Within the predefined subgroups, response rate for EDs increased with their hospital size (Table 1).

**Table 1:** Comparison of participating hospital with the official German hospital directory data [9]

Hospital beds	Total	< 400	400–799	≥ 800
Participating hospitals	147	70	52	25
Proportion of participating hospitals related to study population	**100%**	**47.6%**	**35.4%**	**17.0%**
Number of hospitals in Germany	1,864	1,453	315	96
Proportion of number of hospitals related to total number of hospitals in Germany	**100.0%**	**78.0%**	**16.9%**	**5.1%**
Proportion of participating hospitals related to total number of hospitals in Germany	**7.9%**	**4.8%**	**16.5%**	**26.0%**

Three questionnaires were excluded in the subgroup analysis due to implausible bed counts

### ED structure

ED structure included the following parameters: number of visits, treatment spaces, visits per treatment space, resuscitation beds, visits per resuscitation, observation unit, number of beds in observation unit, visits per observation unit bed, and percentage of cases that arrived by ambulance. Corresponding statistical data are presented in Table 2.

**Table 2:** Emergency department structure parameters

Hospital beds		Total (***n =*** 150)	< 400 (***n =*** 70)	400–799 (***n =*** 52)	≥ 800 (***n =*** 25)
**Number of visits**	valid data (n)	136	61	49	23
	Mean ± SD	29,352 ± 12,275	20,809 ± 8,645	33,740 ± 9,905	41,579 ± 9,358
	Median [IQR]	30,000 [17,008]	20,809 [8,645]	32,840 [9,005]	40,769 [9,838]
	Min–Max	1,450–69,000	1,450–42,000	12,500–64,000	22,000–69,000
**Treatment spaces**	valid data (n)	148	70	51	24
	Mean ± SD	12.8 ± 6.9	9.3 ± 4.5	13.0 ± 5.1	21.7 ± 6.6
	Median	11.0 [8.0]	9.0 [4.0]	12.0 [5.0]	20.0 [10.8]
	Min–Max	2.0–37.0	2.0–28.0	4.0–33.0	11.0–37.0
**Visits per treatment space**	Mean ± SD	2,469 ± 1,038	2,446 ± 1,268	2,714 ± 795	2,083 ± 695
**Resuscitation care spaces**	valid data (n)	149	70	51	25
	Mean ± SD	1.7 ± 0.8	1.3 ± 0.5	1.8 ± 0.7	2.5 ± 1.0
	Median	2.0 [1.0]	1.0 [1.0]	2.0 [1.0]	2.0 [1.0]
	Min–Max	0–5.0	0–3.0	0–4.0	1.0–5.0
**Visits per resuscitation bed**	Mean ± SD	18,822 ± 8,039	17,612 ± 8,696	20,411 ± 7,306	18,251 ± 7,752
**Observation unit (yes)**		83 (56.0%)	23 (32.9%)	37 (71.2%)	22 (88.0%)
**Number of beds in observation unit**	valid data (n)	83	23	36	21
	Mean ± SD	8.8 ± 5.2	6.0 ± 2.4	9.4 ± 5.7	11.1 ± 5.5
	Median	6.0 [4.0]	6.0 [0]	7.5 [4.8]	10.0 [7.5]
	Min–Max	2.0–28.0	2.0–11.0	2.0–28.0	4.0–26.0
**Visits per observation unit bed**	Mean ± SD	4,645 ± 2,283	4,551 ± 2,007	4,644 ± 2,718	4,504 ± 1,841
**Arrived by ambulance (%)**	valid data (n)	96	40	35	18
	Mean ± SD	35.2 ± 14.7	32.1 ± 14.8	34.9 ± 14.3	44.3 ± 11.1
	Median	33.0 [16.1]	30.0 [19.3]	33.0 [14.6]	45.0 [19.8]
	Min–Max	6.0–80.0	7.0–75.0	15.0–80.0	28.0–65.0

Three questionnaires could not be assigned to any subgroup due to implausible bed counts. They were included in the bed-independent analysis under "Total" because plausible data were available for other parameters.

*SD* standard deviation, *IQR* interquartile range, *Min* minimum, *Max* maximum

### ED staffing and governance

A median of 112.8 [IQR: 84.8-131.8] (*n =* 48) clinical care hours were spent by nursing staff, 77.5 [IQR: 47.0-133.7] (*n =* 44) by physician staff, and 25 [IQR: 12.3-35.0] (*n =* 25) by other staff, per 100 cases. This resulted in cumulative clinical care hours of 212.8 hours per 100 cases. The senior decision maker (usually senior specialist or senior physician) had an independent admission right to hospitalize patients in 69.9% (*n =* 146) of the EDs.

### ED population

Participating EDs treated a median of 30,000 [IQR: 20,000-37,008] patients (*n =* 136) (Table 2). Of these patients, a median of 9.8% [IQR: 5.0-15.8%] (*n =* 88) were younger than 19 years and 25.5% [IQR: 20.0-31.6%] were older than 75 years (*n =* 77). A more detailed overview of the patient population is provided in Table 3.

**Table 3:** Emergency department population parameters

Hospital beds		Total (***n =*** 150)	< 400 (***n =*** 70)	400–799 (***n =*** 52)	≥ 800 (***n =*** 25)
**Proportion of patients aged 0–5 years (%)**	valid data (n)	82	38	30	14
	Mean ± SD	3.8 ± 7.5	4.4 ± 10.3	3.2 ± 3.9	3.4 ± 3.2
	Median [IQR]	1.7 [4.0]	1.1 [3.8]	2.0 [4.5]	2.8 [4.0]
	Min–Max	0–62.0	0-62.0	0-14.0	0–10.0
**Proportion of patients aged 0–18 years (%)**	valid data (n)	88	40	31	17
	Mean ± SD	13.5 ± 17.4	14.6 ± 20.9	13.4 ± 16.6	11.0 ± 8.2
	Median [IQR]	9.8 [10.8]	9.3 [8.1]	10.0 [16.8]	9.0 [14.2]
	Min–Max	0–100	0.8-100	0-90.0	1.0–25.0
**Proportion of patients aged over 75 years (%)**	valid data (n)	78	38	27	13
	Mean ± SD	28.0 ± 13.2	28.0 ± 15.8	27.2 ± 6.8	30.0 ± 15.5
	Median [IQR]	25.5 [11.6]	24.9 [13.9]	26.0 [8.1]	25.4 [11.5]
	Min–Max	0–75.0	0-75.0	15.8-48.4	15.0–60.0
**Left without being seen (%)**	valid data (n)	94	35	39	18
	Mean ± SD	1.4 ± 1.4	1.4 ± 1.4	1.6 ± 1.7	1.2 ± 1.2
	Median [IQR]	1.0 [1.5]	1.0 [1.6]	1.0 [1.5]	1.0 [1.2]
	Min–Max	0–7.0	0-5.0	0.1-7.0	0–5.0
**Readmission within 72 hours (%)**	valid data (n)	30	15	7	7
	Mean ± SD	2.1 ± 2.2	2.5 ± 2.7	1.8 ± 1.9	1.8 ± 1.7
	Median [IQR]	1.0 [2.4]	2.0 [3.0]	1.0 [3.7]	1.0 [2.5]
	Min–Max	0–10.0	0-10.0	0-5.0	0.2–5.0

Discrepancies between the sum of subgroups and total resulted from the exclusion of unplausible bed counts as described in Table 2

*SD* standard deviation, *IQR* interquartile range, *Min* minimum, *Max* maximum

**Fig. 1:**
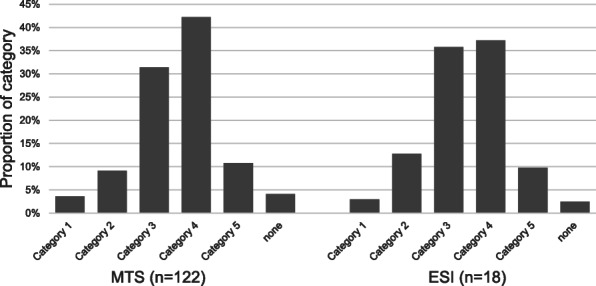
Distribution of initial acuity assesment by Manchester Triage System (MTS) and Emergency Severity Index (ESI)

The initial triage of emergency patients was performed mainly using the Manchester Triage System (MTS) in 122 EDs (81.3%) and the Emergency Severity Index (ESI) in 18 EDs (12.0%). Furthermore, six EDs used proprietary ED-specific initial triage systems (4.0%), three EDs performed no initial triage (2.0%) with one ED not reporting (0.7%). Figure 1 (initial triage – category of acuity for Manchester Triage System (MTS) and Emergency Severity Index (ESI)) shows relative frequencies of each initial triage category for MTS and ESI. Categories three and four were represented most frequently in participating EDs.

### ED performance indicators

The median time until first physician contact was 31.1 [IQR: 17.5-46.0] minutes (*n =* 98). Patients spent a median of 154.0 [IQR: 120.0-192.0] minutes in the ED (*n =* 85). The ED performance indicators are presented in Table 4.

**Table 4:** Performance indicators of emergency department process times

Hospital beds		Total (*n =* 150)	< 400 (*n =* 70)	400–799 (*n =* 52)	≥ 800 (*n =* 25)
Time to first physician contact (min)	valid data	98	45	38	13
	Mean ± SD	33.1 ± 17.9	28.0 ± 14.4	41.3 ± 19.5	31.6 ± 18.6
	Median [IQR]	31.05 [28.5]	29.0 [25.0]	42.5 [23.3]	29.0 [29.3]
	Min–Max	5.0–84.0	5.0-55.0	5.0-84.0	5.0-67.0
Length of stay (min)	valid data	85	39	33	13
	Mean ± SD	170.4 ± 87.8	147.6 ± 87.3	177.0 ± 76.6	221.8 ± 97.3
	Median [IQR]	154.0 [72.0]	140.0 [60.0]	168.0 [73.0]	199.0 [106.5]
	Min–Max	40.0–481.0	40.0-480.0	60.0-464.0	110.0-481.0
Length of stay for inpatient admission (min)	valid data	62	27	22	12
	Mean ± SD	178.5 ± 101.8	132.7 ± 66.1	187.5 ± 90.6	254.4 ± 135.6
	Median [IQR]	173.5 [112.3]	120.0 [90.0]	178.0 [108.0]	238.5 [131.8]
	Min–Max	35.0–530.0	35.0-270.0	50.0-494.0	60.0-530.0
Length of stay for observation unit admissions (min)	valid data	28	8	9	10
	Mean ± SD	388.7 ± 332.5	427.4 ± 431.7	362.3 ± 310.3	353.3 ± 298.1
	Median [IQR]	280.0 [382.5]	315.0 [625.0]	260.0 [510.5]	216.5 [320.0]
	Min–Max	0–1,299.0	30.0-1,299.0	0-938.0	90.0-1,080.0
Length of stay for discharged patients (min)	valid data	58	25	22	11
	Mean ± SD	132.8 ± 41.9	112.4 ± 36.5	138.3 ± 41.6	168.4 ± 26.1
	Median [IQR]	127.5 [52.8]	120.0 [49.0]	139.5 [42.8]	180.0 [48.0]
	Min–Max	50.0–216.0	50.0-198.0	60.0-216.0	120.0-198.0
Length of stay for patients transferred to another hospital (min)	valid data	42	23	14	5
	Mean ± SD	162.5 ± 156.1	108.3 ± 56.6	161.2 ± 92.1	415.6 ± 328.0
	Median [IQR]	120.0 [101.8]	110.0 [60.0]	159.7 [70.8]	300.0 [529.0]
	Min–Max	25.0–938.0	25.0-240.0	50.0-424.0	60.0-938.0

Length of stay is presented separately for each disposition

Discrepancies between the sum of subgroups and total resulted from the exclusion of unplausible bed counts as described in Table 2

*SD* standard deviation, *IQR* interquartile range, *Min* minimum, *Max* maximum

### Hospitals

The participating hospitals had a median of 403 beds (range 127 to 2,058; *n =* 147). A median of 13 [IQR: 10-25] intensive care unit beds (*n =* 144) and 9.5 [IQR: 5.0-16.0] intermediate care beds (*n =* 130) were available.

### ED outcomes

The median proportion of outpatients was 55.2% [IQR: 49.3-63.7%] (*n =* 86). Of the remaining inpatients, 32.3% [IQR: 25.8-40.0%] (*n =* 81) of patients were admitted to a normal care unit, 5.0% [IQR: 2.0-7.1%] (*n =* 74) to an intensive care unit, 6.3% [IQR: 2.0-10.0%] to an observation unit (*n =* 51), 1.2% [IQR: 0.7-3.0%] (*n =* 67) were transferred to another hospital, and 0.1% [IQR: 0.05-0.1%] (*n =* 54) died in the ED.

Patients who left without being seen accounted for 1.0% [IQR: 0.5-2.0%] (*n =* 94). After discharge, 1.0% [IQR: 0.9-3.3%] (*n =* 30) of the patients returned unplanned within 72 hours (Table 2).

## Discussion

This study was the first comprehensive description of the status of EDs in Germany. As no national standard for surveys in EDs existed in Germany, the Utstein reporting standard [11] for research publications was used to collect internationally comparable data. Previous German studies dated back to 2013 were limited to members of ED professional societies and achieved lower respondent numbers [4, 12]. Those studies had a lower response rate of small hospital EDs covering about 9.4% of the study sample [4]. In this study, small hospitals had a better representation with 47.6% but did not reach the proportion in official hospital statistics with 78.0%. Furthermore, representativity assessment was complicated as official hospital statistics contained non-ED hospitals.

Compared to previous publications, EDs in this study had fewer treatment spaces, which was aligned with the higher response rate from smaller hospitals [4, 12].

According to the Federal Joint Committee’s (G-BA) decision from 2018 [13], EDs providing more than basic emergency care are required to have an observation unit. This affects approximately 41% of German EDs [8]. With 56%, the proportion was higher in this sample. However, since there were no further information on the level of care, conclusions about the fulfillment of the G-BA requirements could not be drawn.

Despite low question specific response rate, the estimated direct clinical care hours per patient visit were consistent to previously reported data [4]. This study determined direct clinical care hours, which were on average higher than the patient-dependent engagement times measured by Gräff et al. [14]. These results could not be compared as Gräff et al. measured engagement times using an observer, while this study calculated care hours based on staff roster.

A median of 30,000 patients annually corresponded with 34,000 patients from a previous study with a higher proportion of larger hospitals [4]. Internationally, the number of patient contacts in different healthcare systems differed widely (Switzerland, 8806; United States, 20,000; France, 22,265; and Denmark, 32,000) [15–18].

The proportion of pediatric patients aged 0 to 5 years and 0 to 18 years were higher than those previously reported [4, 19] but were still inadequately represented compared to international data [1, 3, 20]. This may be due to existing specialized pediatric EDs, which were often not organizationally integrated into the EDs and thus not surveyed. Accordingly, patients older than 75 years were overrepresented in this study [1, 3, 16, 20]. In addition to the assumed selection bias by non-participating pediatric EDs, Germany has the fourth oldest population in the world [21], which may have contributed to the increased proportion of older patients with medical conditions.The length of stay (LOS) in this study was shorter, with a median of 154 minutes, compared to 178 minutes in Australia, where a 4-hour rule is in place [2]. Of the 150 responding EDs, only 85 answered the LOS-question. Of these, 16 EDs reported an average LOS under 120 minutes and eight EDs reported an average LOS over 240 minutes. Since recording of discharge or transfer time was not mandatory for billing purposes [10], the reported LOS may not be reliable.

The frequency distributions of the initial triage categories matched a recent analysis conducted by the AKTIN German Emergency Department Data Registry [19]. Small differences may have been due to the larger proportion of patients not assessed in the reference study (approximately 14%) [19]. Compared to Australia and Canada, patients were assessed as being less urgent [1, 2]. This may have been due to the different health care systems and the increasing use of EDs by patients with acute, but non-emergent, treatment demands [22]. In addition, the MTS, which was predominantly used by EDs in this study, tends to underassess older patients, which were also highly represented in the study [23, 24]. The initial triage of patients as more urgent was more frequent with ESI than with MTS. This could be explained by the fact that MTS and ESI use different algorithms and ESI allows urgent grading based on condition, symptoms, or a combination of both [24].

The outpatient proportion was congruent with previous German results [4] but lower than internationally reported proportions [1, 3, 16, 20]. This may have been due to differences in the health care systems and the higher proportion of elderly patients in this survey data. Furthermore, there is no specialist in emergency medicine in Germany, and EDs are often staffed by younger residents [4] who may make different decisions compared to experienced specialists. Internationally, Germany had one of the highest hospital bed densities [25]. At the same time, EDs do not cover their costs [26]. There is a lack of cost-covering billing numbers in Germany for outpatient emergency care. This applies, for example, to the reimbursement of complex diagnostics in order to avoid admissions. There is an urgent requirement for adequate compensation to avoid false incentives to cover costs through patient hospitalization.

Overall, the survey revealed further research requirements.

### Limitations

Although the overall response rate was good (19.8%), this study covered only a small proportion of German EDs (14.1%). This discrepancy resulted from the fact that no official directory existed and not all German EDs were included in the used directory. Response rate related to hospital size was presumably higher in larger hospitals. Furthermore, while the overall item-specific response rates were acceptable, some case-related questions (e.g. process times, and disposition) suffered relevant omissions. There was no feedback with respect to the motivation not to respond to the survey. Due to the structure of the Utstein template, the evaluation of time to physician treatment related to triage category was not possible. In future surveys, the classification of hospitals into the new G-BA national emergency levels [13] should be recorded for better evaluability.

## Conclusion

This study enabled, for the first time, a nationwide survey addressing individual EDs in terms of structure, key figures, and performance indicators. Politicians and healthcare managers may use these data for further planning and development in clinical emergency medicine. To be more representative and to allow regional planning, the collection of these data should become mandatory for all German EDs.

## Abbreviations

ED: Emergency department
SD: Standard deviation
IQR: Interquartile range
DIVI: German interdisciplinary association of critical care and emergency medicine
DGINA: German interdisciplinary society for emergency and acute medicine
MTS: Manchester triage system
ESI: Emergency severity index
LOS: Length of stay
